# Retraction: miR-517a is upregulated in glioma and promotes glioma tumorigenesis in vitro and in vivo

**DOI:** 10.1042/BSR-2018-1196_RET

**Published:** 2023-01-16

**Keywords:** glioma, invasion, migration, miR-517a, proliferation

This Retraction follows an Expression of Concern relating to this article previously published by Portland Press. This article is being retracted from *Bioscience Reports* at the request of the Editor-in-Chief and the Editorial Board following receipt of a notification from a reader, alerting the Editorial Board to similarities between images in the figures and those of other articles from unrelated authors. The tumour images in Figure 6A share strong similarities with images in both Figure 4 from Sun et al. 2016 (DOI: 10.1007/s13277-016-5444-9) and Figure 3A by Kong et al. 2015 (DOI: 10.1186/s12943-015-0355-8). The cell images in Figure 6D appear, rotated 90 degrees, in Figure 5D from Han et al. 2015 (DOI: 10.1038/cddis.2015.30), and the western blots of figure 5B appear very similar to those in the same paper by Han et al (2015) in Figure 6C. The authors have been contacted with regards to the retraction and have not responded to the Journal's queries or the concerns raised. Given the extent of the issues raised, the Editorial Board stand by the decision to retract the article.

